# DEMENTIA CARE IN NURSE PRACTITIONER-LED CARE MANAGEMENT FOR COGNITIVELY VULNERABLE OLDER ADULTS

**DOI:** 10.1093/geroni/igac059.1008

**Published:** 2022-12-20

**Authors:** Richard Fortinsky, Shawn Ladda

**Affiliations:** University of Connecticut Center on Aging, Farmington, Connecticut, United States; UConn Center on Aging, University of Connecticut School of Medicine, Farmington, Connecticut, United States

## Abstract

Care management approaches are being widely tested in the Medicare-eligible population to manage chronic conditions, but few have focused on cognitive vulnerability as the pathway to optimizing independence in the community-dwelling older population. Cognitive vulnerability refers to living with dementia, depression, and/or a history of delirium. This presentation features a nurse practitioner-led team care management model (3D Team) to address cognitive vulnerability, tested in an ongoing clinical trial with older adults in a Medicare Advantage population. For older adults with dementia and their families served by the 3D Team, the nurse practitioner works closely with occupational therapists (OTs) delivering a nonpharmacological dementia care intervention. Preliminary results presented will include: characteristics of dyads that have received the dementia care intervention (N=70 dyads to date), how the nurse practitioner and OTs communicate, how the nurse practitioner reinforces dementia care skill-building strategies introduced by OTs, and process evaluation results to date.

